# Scrub typhus triggers hemophagocytic lymphohistiocytosis in children: metagenomic next-generation sequencing enables rapid diagnosis

**DOI:** 10.3389/fpubh.2026.1888645

**Published:** 2026-09-16

**Authors:** Lin Qihong, Wang Juan, Tang Xue, Ding Zhiheng, Chen Dongyan, Yang Lili, Wen Feiqiu, Liu Sixi, Liu Shilin

**Affiliations:** Department of Hematology and Oncology, Shenzhen Children's Hospital, Shenzhen, Guangdong, China

**Keywords:** cerebrospinal fluid, hemophagocytic lymphohistiocytosis, metagenomic next-generation sequencing, pediatrics, pulmonary hemorrhage, scrub typhus

## Abstract

**Background:**

Hemophagocytic lymphohistiocytosis (HLH) is a life-threatening hyperinflammatory syndrome. Rapid identification of the underlying trigger is critical for effective clinical management. This study describes the clinical features and diagnostic approach for scrub typhus-associated HLH in children, emphasizing the role of metagenomic next-generation sequencing (mNGS).

**Methods:**

We retrospectively analyzed 12 pediatric patients diagnosed with scrub typhus-associated HLH at Shenzhen Children’s Hospital between July 2023 and July 2025. Demographic, clinical, laboratory, and treatment data were analyzed. The diagnostic timeline was systematically evaluated, including patient’s delay, doctor’s delay, and mNGS turnaround time.

**Results:**

The median age was 7.5 years (range: 3–13.7), with a male-to-female ratio of 1:2. All patients presented with prolonged fever (median duration: 9 days). Common clinical findings included eschar (66.7%), lymphadenopathy (100%), hepatomegaly (58.3%), and splenomegaly (66.7%). Notably, four patients (33.3%) were initially misdiagnosed as having Kawasaki disease. All patients met the HLH-2004 criteria, with prominent hyperferritinemia (median: 5,355 ng/mL) and elevated sCD25 (median: 5,065 U/mL). Peripheral blood mNGS confirmed the diagnosis of scrub typhus in all patients (median sequence reads: 333). Cerebrospinal fluid (CSF) mNGS was positive for *Orientia tsutsugamushi* in four out of five patients tested, and cytokine profiling revealed elevated levels of IL-6, IL-10, and IFN-γ. Basic CSF parameters, including white blood cell count, protein, and glucose, are reported for all patients who underwent lumbar puncture. Severe complications included pulmonary hemorrhage (*n* = 3) and shock (*n* = 3). All patients survived following targeted antibiotic and immunomodulatory therapy.

**Conclusion:**

Scrub typhus is a significant trigger for secondary HLH in children from endemic areas. mNGS may facilitate rapid etiological diagnosis and timely targeted therapy. CSF mNGS and cytokine analysis may be considered in severe cases, though further validation is warranted. We also acknowledge that our study demonstrates feasibility rather than superiority of mNGS over conventional diagnostic methods.

## 1 Introduction

Hemophagocytic lymphohistiocytosis (HLH) is a severe, life-threatening syndrome of excessive immune activation, frequently leading to multi-organ failure and high mortality if not promptly treated (1). HLH is classified into primary (familial) and secondary forms. Secondary HLH is commonly triggered by infections, malignancies, or autoimmune diseases (2). Among infectious triggers, viral pathogens like Epstein–Barr virus and bacterial infections such as typhoid are well-recognized (3, 4). In endemic areas, rickettsial infections, particularly scrub typhus caused by *Orientia tsutsugamushi*, represent a significant cause of infection-associated HLH (5).

Scrub typhus is an acute febrile illness transmitted through the bite of infected chiggers. After an incubation period of 5–14 days, patients typically present with fever, headache, myalgia, rash, and often a characteristic eschar at the inoculation site (6–8). Severe complications can include hepatitis, pneumonia, meningitis, shock, multi-organ dysfunction syndrome (MODS), and progression to HLH (5, 9–11).

The non-specific clinical presentation of scrub typhus and the diverse etiology of HLH pose considerable diagnostic challenges (1, 12). In endemic regions such as southern China, the seasonal peak of scrub typhus (April–October) coincides with increased outdoor activities, necessitating heightened clinical awareness (13). Regional epidemiological surveillance in nearby Guangzhou has identified the Karp genotype as the predominant circulating strain, which has been associated with more severe disease manifestations including longer hospital stays and higher rates of organ involvement (14). Consequently, the rapid identification of scrub typhus as the underlying trigger for HLH is paramount. This study reports 12 pediatric cases of scrub typhus-associated secondary HLH, highlighting the utility of rapid mNGS for early etiological diagnosis and providing detailed epidemiological and clinical characterization.

Compared with previous pediatric case series, our study is distinguished by: (i) systematic dissection of diagnostic delays, including patient- and doctor-related intervals; (ii) combined application of CSF mNGS and CSF cytokine profiling to characterize CNS involvement; and (iii) provision of denominator data on scrub typhus admissions, enabling preliminary estimation of HLH incidence in this population.

## 2 Materials and methods

### 2.1 Study population and design

We retrospectively reviewed the medical records of 12 pediatric patients diagnosed with scrub typhus-associated HLH at Shenzhen Children’s Hospital between July 2023 and July 2025. During the study period, a total of 22 pediatric patients were diagnosed with scrub typhus at our center. Among these, 12 patients (54.5%) met the HLH-2004 diagnostic criteria and were included in this case series. The remaining 10 scrub typhus patients without HLH did not undergo mNGS, as they were diagnosed clinically and responded to empirical antibiotics. The study was approved by the hospital’s Ethics Committee, and the legal guardians of all participants provided written informed consent.

### 2.2 Diagnostic criteria

The diagnosis of HLH was established based on the HLH-2004 diagnostic criteria (1), requiring the fulfillment of at least 5 out of the following 8 criteria: (1) Fever, (2) Splenomegaly, (3) Cytopenias (affecting ≥2 of 3 lineages: hemoglobin <90 g/L, neutrophils <1.0 × 10^9^/L, and platelets <100 × 10^9^/L in peripheral blood), (4) Hypertriglyceridemia (fasting triglycerides ≥3 mmol/L) and/or hypofibrinogenemia (fibrinogen ≤1.5 g/L), (5) Hemophagocytosis in bone marrow, spleen, or lymph nodes, (6) Low or absent NK-cell activity, (7) Hyperferritinemia (ferritin ≥500 ng/mL), (8) Elevated soluble CD25 (sIL-2R alpha chain ≥2,400 U/mL). Scrub typhus was confirmed by detecting *Orientia tsutsugamushi* sequences via mNGS of peripheral blood and/or cerebrospinal fluid (CSF). In this retrospective cohort, mNGS of peripheral blood was the sole microbiological confirmation method for scrub typhus. Conventional serological tests (Weil-Felix, IgM ELISA, IFA) and PCR were not performed systematically as part of routine clinical care during the study period, as mNGS was increasingly adopted as a first-line diagnostic tool for unexplained febrile illnesses in our center.

### 2.3 mNGS methodology

DNA was extracted from peripheral blood and cerebrospinal fluid (CSF) samples using a commercial DNA extraction kit. Subsequently, sequencing libraries were constructed following standard protocols. For bioinformatic analysis, human-derived sequences were first filtered out, and the remaining microbial reads were aligned against reference databases to identify potential pathogens.

### 2.4 Data collection

For each patient, we collected demographic information, clinical features, laboratory results (including complete blood count, coagulation profile, ferritin, fibrinogen, triglycerides, sCD25, and cytokine levels), imaging findings, treatment details, and clinical outcomes. Detailed diagnostic timeline data were systematically recorded, including: (1) date of symptom onset; (2) date of first medical consultation; (3) date of mNGS sampling; (4) date of mNGS result reporting; (5) date of correct diagnosis; and (6) date of targeted therapy initiation. Exposure history, including outdoor activities, rural residence, travel history, and presumed locations of infection acquisition, was collected when available.

### 2.5 Statistical analysis

Descriptive statistics were used. Continuous variables were presented as medians with ranges or interquartile ranges (IQR), and categorical variables as counts and percentages.

## 3 Results

### 3.1 Patient characteristics and clinical presentation

The study included 12 pediatric patients (4 males, 8 females) with a median age of 7.5 years (range: 3–13.7 years). These 12 patients were derived from a total of 22 scrub typhus admissions during the same period, representing an HLH incidence of 54.5% among hospitalized pediatric scrub typhus patients. All cases occurred between April and October, aligning with the peak activity period of chiggers in endemic regions, which underscores the seasonality of scrub typhus transmission. Specifically, cases were distributed as follows: April (*n* = 1), May (*n* = 1), June (*n* = 2), July (*n* = 5), and October (*n* = 3). Regarding exposure history, 10 patients (83.3%) resided in rural or suburban areas of Shenzhen, 8 patients (66.7%) reported recent outdoor activities (e.g., farming, hiking, or playing in grassy areas) within 2 weeks prior to symptom onset, and 2 patients (16.7%) had travel history to other endemic regions in Guangdong Province. No occupational exposures among household members were identified. The median duration of fever before admission was 9 days (range: 7–13 days), highlighting the prolonged febrile course commonly associated with scrub typhus and the potential delay in seeking specialized care or establishing a correct diagnosis.

Common clinical findings at diagnosis included universal presence of fever (100%) and lymphadenopathy (100%), hepatosplenomegaly (hepatomegaly 58.3%, splenomegaly 66.7%), and eschar (66.7%). The eschar (Figure 1), a pathognomonic sign, was not always immediately evident, requiring meticulous physical examination. Notably, four patients (33.3%) were initially suspected to have Kawasaki disease due to overlapping features such as persistent fever, rash, and conjunctival injection, underscoring the significant diagnostic challenge and potential for misdiagnosis in the early stages. Patient characteristics are comprehensively summarized in Table 1 and Table 2. Baseline characteristics of the 10 scrub typhus patients without HLH are provided in Supplementary Table S1.

**Figure 1:**
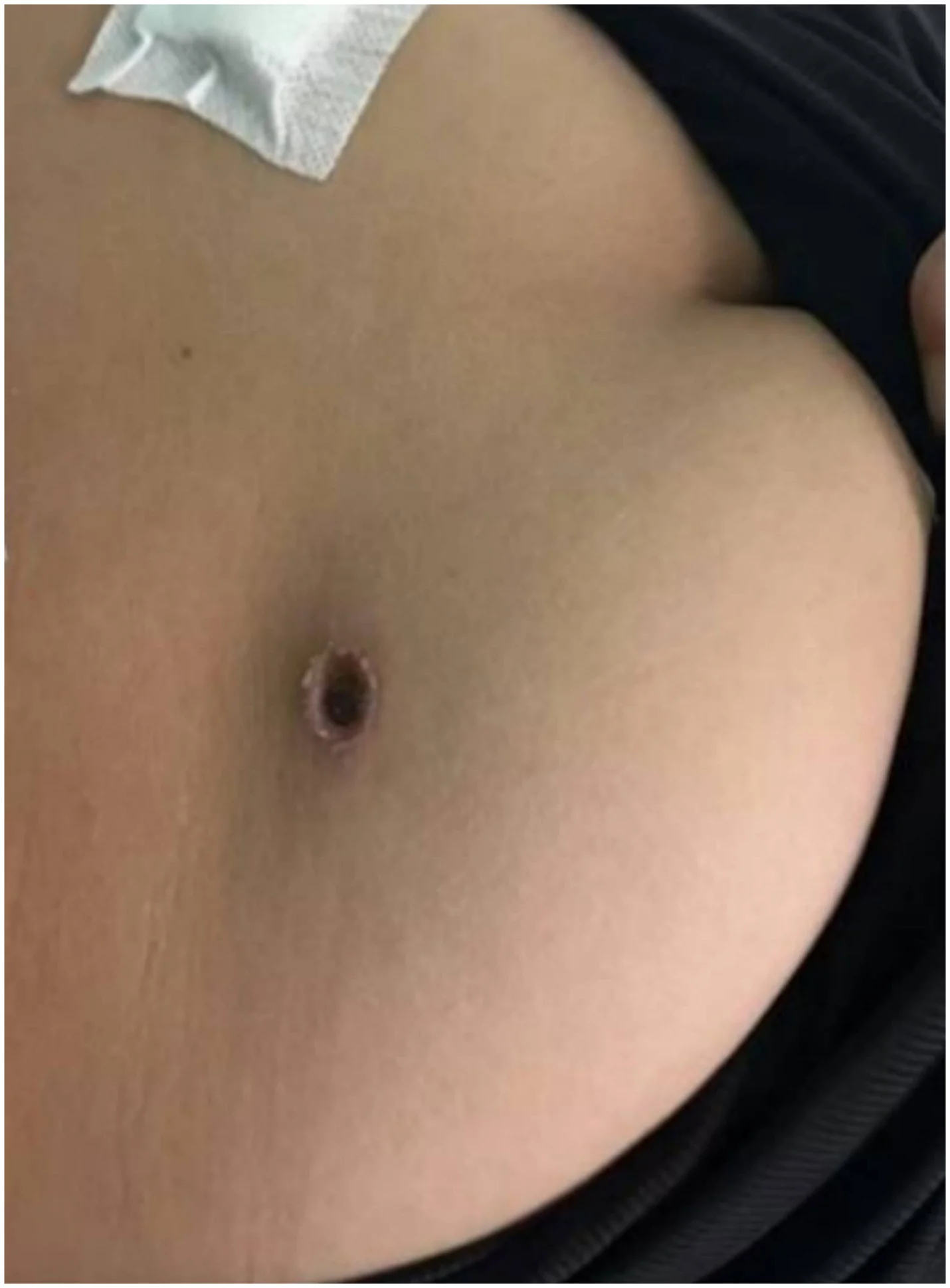
A necrotic eschar identified in the vicinity of the left buttock of case 9. This lesion was not identified on initial physical examination, highlighting the importance of meticulous skin inspection in febrile children with suspected scrub typhus. The eschar, a pathognomonic sign, can be easily overlooked in skin folds or pigmented skin, contributing to diagnostic delays.

**Table 1:** Clinical demographics and diagnostic timeline.

Patient no.	Age	Gender	Time of diagnosis	Admitting diagnosis	Days of diagnosis in admission (ST-HLH)
1	4y7m	M	July	Pneumonia	3
2	4y3m	F	October	Pneumonia	5
3	3y5m	F	October	Kawasaki disease	5
4	3y	F	October	Pneumonia	3
5	4y11m	F	April	Pneumonia	5
6	10y2m	M	June	Kawasaki disease	4
7	3y8m	F	July	Pneumonia	3
8	11y8m	F	May	Kawasaki disease	4
9	11y3m	F	June	Pneumonia	2
10	10y6m	M	July	Kawasaki disease	2
11	9y7m	F	July	Pneumonia	3
12	13y7m	M	July	Kawasaki disease	4

**Table 2:** Clinical and laboratory findings at HLH diagnosis.

Patient no.	Days of fever duration	Rash/Eschar	Lymphadenopathy/ Hepatomegaly/ Splenomegaly	HB(g/L)/ ANC (x10^9^/L)/ PLT (x10^9^/L)	Fibrinogen (g/L)/ TG(mmol/L)	Ferritin (ng/mL)/sCD25(U/mL)	Hemophagocytosis (BM)
1	10	−/−	+/+/+	93/1.53/31	2.03/5.3	1,218/1,923	+
2	10	−/−	+/+/+	70/2.6/22	0.8/3.72	29,913/12,736	+
3	7	+/−	+/+/+	79/3.92/69	1.31/6.5	11,408/3,511	+
4	8	+/+	+/−/−	98/4.54/50	1.06/5.02	10,152/5,301	+
5	7	−/−	+/+/+	78/2.11/51	0.9/7.26	6,340/7,953	+
6	10	+/−	+/−/−	103/1.11/75	1.38/2.71	13,315/3,521	+
7	10	+/−	+/−/+	86/4.28/63	1.38/3.41	1,713/609	/
8	9	+/+	+/−/+	105/1.45/63	2.35/1.58	3,209/4,957	+
9	7	+/+	+/−/−	98/1.74/99	3.0/1.98	602/2,523	+
10	8	+/+	+/+/+	83/4.48/63	0.52/3.32	5,355/6,598	+
11	11	+/+	+/−/−	104/2.4/66	1.38/3.27	1,331/5,172	+
12	13	+/+	+/−/−	111/3.14/94	1.93/3.87	4,501/6,128	+

HB, Hemoglobin; ANC, Absolute Neutrophil Count; PLT, Platelets; TG, Triglycerides; sCD25, Soluble CD25; BM, Bone Marrow.

### 3.2 Laboratory findings

Key laboratory findings consistent with HLH are summarized in Table 2. All patients exhibited cytopenias affecting at least two lineages, a hallmark of HLH reflecting bone marrow suppression or peripheral destruction. Hemoglobin levels were below 90 g/L in 5 patients (41.7%), indicating significant anemia, and all patients had thrombocytopenia (platelets <100 × 10^9^/L), which contributed to bleeding tendencies.

Coagulation abnormalities were prominent: hypofibrinogenemia (<1.5 g/L) was present in 8 patients (66.7%), and hypertriglyceridemia (≥3 mmol/L) was observed in 9 patients (75%), both key metabolic disturbances in HLH. A striking finding was the significant hyperferritinemia in all patients (median 5,355 ng/mL, range: 602–29,913), with levels vastly exceeding the diagnostic threshold, underscoring its role as a key indicator of the severe hyperinflammatory state. Hemophagocytosis was observed in the bone marrow of all 11 patients who underwent aspiration, providing crucial histological confirmation of HLH. Soluble CD25 levels, reflecting T-cell activation, were elevated (>2,400 U/mL) in 10 out of 12 patients (83.3%), further supporting the diagnosis of immune dysregulation.

### 3.3 Diagnostic timeline and mNGS performance

The diagnostic timeline analysis revealed the following: the median time from symptom onset to first medical consultation (patient’s delay) was 4 days (IQR: 3–5 days). The median time from first medical consultation to correct diagnosis (doctor’s delay) was 5 days (IQR: 3–7 days). mNGS demonstrated a median turnaround time of 48 h (IQR: 36–72 h) from sample collection to result reporting. The median time from mNGS sampling to diagnosis confirmation was 2 days (IQR: 1.5–3 days).

All patients tested positive for *Orientia tsutsugamushi* via peripheral blood mNGS, with a wide range of sequence reads (median 333, IQR: 256–874). This confirmed the etiology and demonstrated the feasibility of mNGS as a rapid, sensitive, and unbiased diagnostic tool in this clinical context. Although genotypic analysis of the mNGS sequences was not feasible due to the limited read length and coverage depth, the geographic distribution of cases (all within Shenzhen and surrounding areas) suggests exposure to locally circulating strains.

It is important to note that our study demonstrates feasibility and potential utility, but it does not establish superiority over conventional methods. A direct comparative study with PCR or serology would be required to make such claims.

### 3.4 CSF findings and neurological involvement

Five patients underwent lumbar puncture and CSF mNGS analysis. Basic CSF parameters for these patients are summarized in Table 3. CSF white blood cell counts ranged from 5 to 245 cells/μL (median: 68 cells/μL) with lymphocytic predominance (65–90%). CSF protein concentrations were elevated in all five patients, ranging from 0.56 to 1.89 g/L (median: 1.12 g/L). CSF glucose levels were normal or mildly decreased (median: 2.8 mmol/L, range: 2.1–3.6 mmol/L). Opening pressure was measured in three patients and was elevated (>200 mmH₂O) in two patients with overt neurological symptoms.

**Table 3:** Neurological manifestations, CSF parameters and mNGS findings.

Patient no.	Neurological symptoms	CSF WBC (cells/μL)/ CSF Lymphocyte (%)	CSF Protein (g/L)/ CSF Glucose (mmol/L)	Opening Pressure (mmH₂O)	CSF IL-6 (pg/mL)/IL-10 (pg/mL)/IFN-γ (pg/mL)	Peripheral Blood mNGS (reads)	CSF mNGS (reads)
8	Dizziness, neck stiffness	245/90	1.89/2/1	280	141.62/36.79/13.1	333	6
9	–	8/65	0.56/3/6	–	28.23/8.25/4.49	73	4
10	Irritability, lethargy	128/85	1.56/2.4	220	183.21/31.54/13.24	874	49
11	–	5/70	0.68/3/2	–	10.42/9.32/15.23	256	0
12	–	9/75	0.88/2.8	–	20.39/24.46/12.57	6,799	79

CSF, Cerebrospinal fluid; IL, Interleukin; IFN-γ, Interferon-gamma; mNGS, Metagenomic next-generation sequencing.

*Orientia tsutsugamushi* was detected in the CSF of four of these patients, all of whom presented with overt neurological symptoms such as dizziness, irritability, lethargy, and neck stiffness. This direct pathogen detection in the CSF confirms central nervous system (CNS) invasion and establishes a definitive diagnosis of rickettsial meningoencephalitis.

Cytokine analysis in the CSF revealed notably elevated levels of IL-6, IL-10, and IFN-γ in these patients (Table 3), suggesting intense neuroinflammation driven by the cytokine storm characteristic of HLH. Interestingly, one patient (Case 9) without overt neurological symptoms also showed elevated CSF cytokines and a positive CSF mNGS, indicating possible subclinical CNS involvement that might be more common than previously recognized. Another patient (Case 11) had elevated CSF IFN-γ despite negative CSF mNGS, highlighting the complementary roles of pathogen detection and immune response profiling. Including the five patients who underwent CSF analysis, all presented with neurological signs or severe systemic illness, which guided the decision to perform lumbar puncture.

### 3.5 Treatment, complications, and outcomes

All patients received targeted anti-rickettsial antibiotics (doxycycline and/or azithromycin), which form the cornerstone of etiological treatment. This was combined with immunomodulatory therapy for HLH, including corticosteroids at a median starting dose of 2 mg/kg/day of methylprednisolone (range: 1.5–4 mg/kg/day), with a median total duration of 14 days (range: 10–28 days), followed by a tapering course over 2–4 weeks, intravenous immunoglobulin (IVIG), and/or the JAK–STAT pathway inhibitor ruxolitinib, tailored to disease severity.

The management of these complex cases was challenging due to severe complications. Three patients (Cases 2, 5, 10) developed life-threatening pulmonary hemorrhage (Figure 2). All three had profound thrombocytopenia and evidence of coagulopathy, suggesting a synergy between endothelial damage from the infection and HLH-associated clotting dysfunction leading to diffuse alveolar hemorrhage. Two of these patients (Cases 2 and 5) required invasive mechanical ventilation due to respiratory failure, indicating the critical nature of this complication. Case 8 was diagnosed with rickettsial encephalopathy, demonstrating the potential for severe neurological sequelae. Furthermore, three patients (Cases 2, 5, 12) experienced shock, emphasizing the systemic severity and the potential for multi-organ dysfunction.

**Figure 2:**
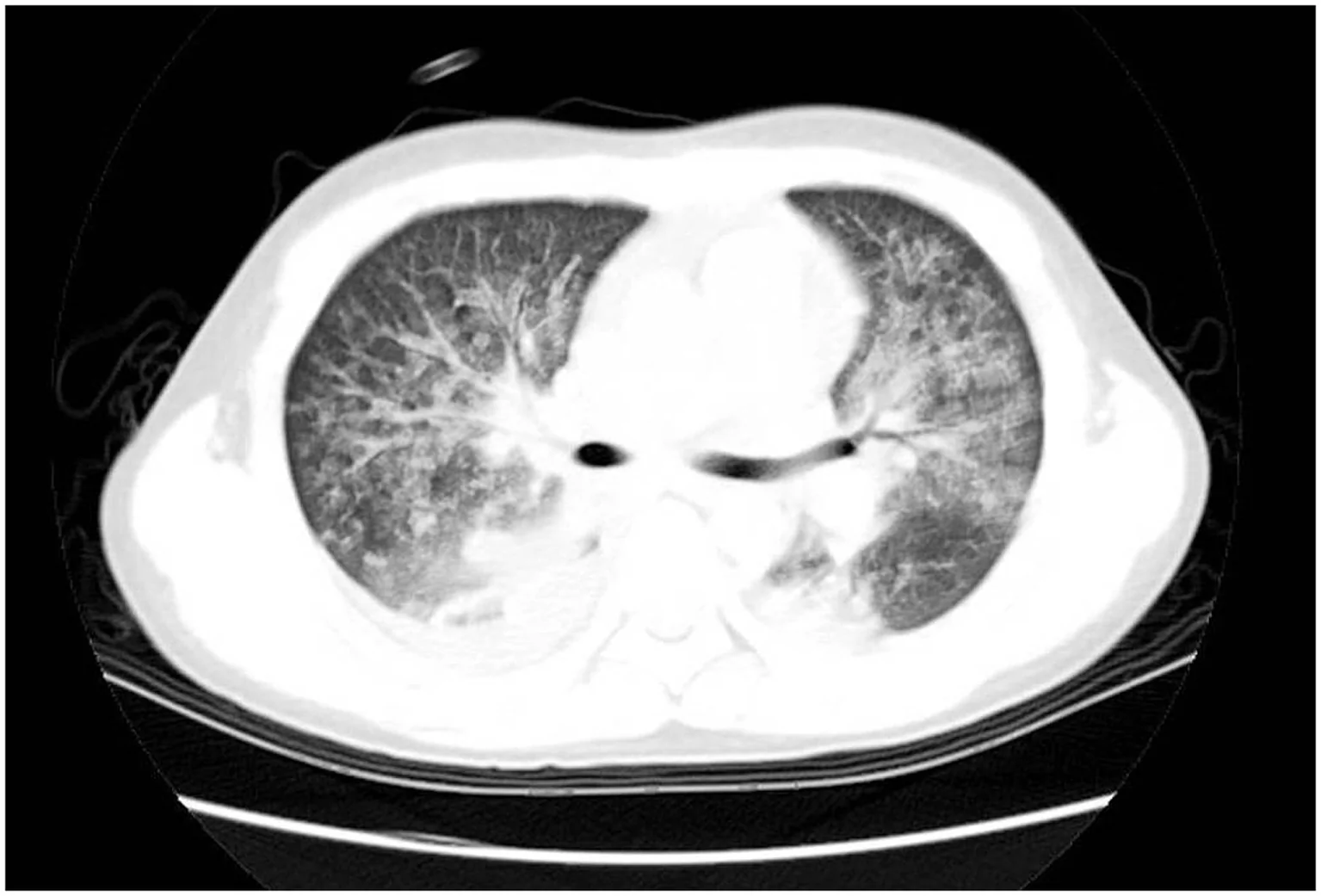
Chest CT scan of case 10 showing bilateral pulmonary hemorrhage with ground-glass opacities and consolidation, demonstrating bilateral pulmonary hemorrhage with diffuse ground-glass opacities and consolidation. This patient presented with profound thrombocytopenia (platelets 63 × 10^9^/L) and severe coagulopathy (fibrinogen 0.52 g/L), requiring invasive mechanical ventilation. The imaging findings illustrate the life-threatening pulmonary complication that can arise from the combined effects of endothelial damage from *Orientia tsutsugamushi* and the hyperinflammatory cytokine storm of HLH.

Despite these severe and potentially fatal complications, all patients survived, which is a testament to the importance of rapid etiological diagnosis via mNGS, timely initiation of appropriate antibiotics, and aggressive supportive care combined with immunomodulation. Detailed treatment regimens and complications for each patient are provided in Table 4.

**Table 4:** Treatment, complications, and outcomes.

Patient no.	Anti-rickettsial antibiotics	Other drugs	Complications
1	Azithromycin	Ruxolitinib, Cefoperazone sulbactam	–
2	Doxycycline, Azithromycin	Ruxolitinib, Vancomycin, Cefoperazone sulbactam	Shock, Pulmonary hemorrhage, Severe pneumonia (Mechanical ventilation)
3	Doxycycline	/	–
4	Azithromycin	Vancomycin, Cefoperazone sulbactam	–
5	Azithromycin	Ruxolitinib, Cefoperazone sulbactam	Shock, Pulmonary hemorrhage, Severe pneumonia (Mechanical ventilation)
6	Doxycycline	Ruxolitinib	–
7	Azithromycin	Ruxolitinib	–
8	Doxycycline	Ruxolitinib, Vancomycin, Cefoperazone sulbactam, Mannitol	Rickettsia encephalopathy
9	Doxycycline	/	–
10	Doxycycline, Azithromycin	Ruxolitinib, Fluconazole, Cefoperazone sulbactam	Pulmonary hemorrhage Severe pneumonia
11	Doxycycline	/	–
12	Doxycycline	Meropenem	Shock, Pneumonia

All patients survived.

## 4 Discussion

This case series highlights the diagnostic challenge and severe clinical course of scrub typhus-associated HLH in children. The initial clinical presentation, particularly fever and rash, can mimic other conditions like Kawasaki disease, as seen in four of our patients. This overlap underscores the importance of meticulous physical examination to uncover often-hidden eschars and a high index of suspicion during peak transmission seasons (April–October) for early recognition of scrub typhus in febrile children with suspected HLH (6, 12). The high misdiagnosis rate in our cohort (33.3%) aligns with literature emphasizing the non-specific early symptoms of scrub typhus (8). The seasonal distribution of our cases (July peak, *n* = 5) is consistent with the known epidemiological pattern of scrub typhus in southern China (13), where increased rainfall and humidity during the summer months favor chigger proliferation.

Although not the primary focus, the descriptive comparison with the 10 scrub typhus patients without HLH (Supplementary Table S1) suggests that patients who developed HLH had a longer fever duration (9 vs. 5 days) and a higher prevalence of hepatosplenomegaly (splenomegaly: 66.7% vs. 30.0%; hepatomegaly: 58.3% vs. 20.0%). This raises the hypothesis that prolonged untreated infection and intense reticuloendothelial system involvement may predispose to HLH, warranting future prospective investigation.

While diagnosing HLH based on established criteria is relatively straightforward, rapidly identifying its trigger is critical for initiating targeted therapy (15). Traditional serological tests for scrub typhus, such as the Weil-Felix test and indirect immunofluorescence assay (IFA), have limitations in sensitivity, especially during the acute phase (16). In contrast, our study demonstrates the pivotal role of mNGS. By providing a rapid, sensitive, and specific etiological diagnosis directly from peripheral blood, mNGS was associated with a median time of 4 days from first consultation to definitive diagnosis in our cohort. However, we emphasize that this observation reflects feasibility, not comparative superiority, as no concurrent serological or PCR testing was performed. Specifically, the median mNGS turnaround time of 48 h in our cohort is substantially shorter than the time required for seroconversion in traditional serological assays, which typically require 7–14 days from symptom onset. This rapid diagnostic capability is particularly valuable in critically ill HLH patients were delayed treatment is associated with increased mortality. This aligns with the growing evidence supporting mNGS for diagnosing obscure and critical infections (17–19). However, it is important to note that the use of mNGS is constrained by higher costs and the potential for environmental contamination and thus should be interpreted in conjunction with clinical findings. Critically, once scrub typhus was identified as the trigger of HLH, targeted anti-rickettsial antibiotics enabled effective control of the infection, reducing the antigenic drive for the hyperinflammation. This etiologic treatment is a cornerstone of managing infection-triggered HLH and may reduce the need for prolonged or more intensive immunosuppressive regimens. Our study does not include a comparator group, and thus we cannot conclude that mNGS is superior to conventional diagnostics. Future prospective comparative studies are needed.

The pathogenesis of neurological involvement in scrub typhus is multifactorial (20–22). *Orientia tsutsugamushi* primarily infects vascular endothelial cells (23). The dense vasculature of the brain makes it a potential target (24). Direct invasion and damage to cerebrovascular endothelial cells compromise the blood–brain barrier (BBB) (25). Furthermore, the systemic hyperinflammatory state in HLH, characterized by a cytokine storm (e.g., high levels of IL-6, IL-10, IFN-γ), can further increase BBB permeability, allowing inflammatory cells and cytokines to infiltrate the central nervous system (26). In our study, CSF mNGS detected *Orientia tsutsugamushi* in four patients, and elevated CSF cytokines were observed even in one patient (Case 9) without overt neurological symptoms. The CSF profiles showed lymphocytic pleocytosis and elevated protein in all five patients who underwent lumbar puncture, consistent with the typical findings of rickettsial meningoencephalitis. The two patients with elevated opening pressure and more pronounced CSF abnormalities (Cases 8 and 10) had the most severe clinical presentations, suggesting a correlation between CSF findings and disease severity. This suggests subclinical CNS involvement might be more common than previously recognized. Therefore, we suggest that early lumbar puncture with CSF mNGS and cytokine analysis may be considered in children with scrub typhus-associated HLH who present with neurological signs or severe systemic illness, though larger studies are needed to establish routine recommendations. Our recommendation is deliberately cautious, given the small sample size and retrospective design.

Pulmonary hemorrhage was a life-threatening complication observed in three patients, all of whom had profound thrombocytopenia and coagulopathy. The potential mechanisms likely involve a triad of factors: (1) direct endothelial damage by the pathogen leading to vasculitis, (2) immune-mediated vascular injury from the HLH cytokine storm, and (3) coagulopathy from HLH-associated disseminated intravascular coagulation (DIC) (27). However, it is important to emphasize that these mechanistic interpretations are based on indirect evidence and previous literature; direct evidence from our cohort is limited. Acknowledging this limitation, the observed combination of severe thrombocytopenia and coagulopathy likely predisposes to diffuse alveolar hemorrhage. Vigilant monitoring of platelet counts, coagulation parameters (especially fibrinogen, D-dimer, PT/APTT), and respiratory status is imperative in severe scrub typhus. Proactive measures such as timely platelet transfusion, correction of coagulopathy (e.g., with cryoprecipitate for hypofibrinogenemia), and consideration of adjunctive therapies to modulate endothelial dysfunction should be considered to prevent this fatal complication (28, 29).

The use of immunomodulatory therapy in our cohort, including corticosteroids, IVIG, and the JAK–STAT inhibitor ruxolitinib, was tailored to disease severity. Ruxolitinib, by inhibiting key cytokines (e.g., IFN-γ, IL-6) involved in the HLH pathogenesis, offers a targeted approach to dampen the hyperinflammatory cascade (15). Its successful use in several of our severe cases, alongside antibiotics, highlights a potential therapeutic strategy for managing the immune dysregulation in infection-triggered HLH while combating the underlying pathogen. However, the optimal timing and combination of these agents in scrub typhus-HLH require further study.

This study also makes a novel contribution by systematically evaluating diagnostic timelines, including patient’s delay, doctor’s delay, and mNGS turnaround time. Our data suggest that mNGS may reduce doctor’s delay and enables prompt targeted therapy. However, patient-related delays remain a substantial challenge, highlighting the need for public health education in endemic areas regarding early recognition of scrub typhus.

### 4.1 Limitations

This study has several limitations, including its retrospective, single-center design and the small sample size, which is inherent to the rarity of this condition. Not all patients underwent uniform diagnostic testing, such as CSF mNGS or NK-cell activity assays, which may introduce some selection bias. Furthermore, mNGS was the sole microbiological confirmation method in this cohort; conventional serology and PCR were not systematically performed. This limits our ability to compare diagnostic performance across different testing modalities.

The inclusion of only mNGS-tested patients introduces selection bias, and our findings may not be generalizable to all scrub typhus-HLH patients. The denominator data (22 total scrub typhus admissions) are provided to contextualize the cohort, but prospective validation is needed.

The absence of a systematic comparison group of scrub typhus patients without HLH precludes the identification of specific risk factors for HLH development. Although we provide descriptive data on the 10 non-HLH scrub typhus patients in Supplementary Table S1, this comparison was not the primary focus of this retrospective study.

Additionally, mNGS, while sensitive, is associated with higher costs and potential for environmental contamination. Genotypic analysis of the *Orientia tsutsugamushi* strains could not be performed due to limitations in mNGS read length and coverage depth, which limits our epidemiological characterization.

## 5 Conclusion

In conclusion, scrub typhus is an important trigger for secondary HLH in children from endemic areas. mNGS is a promising diagnostic tool that can facilitate the rapid identification of *Orientia tsutsugamushi*, supporting prompt and targeted treatment. However, its superiority over conventional methods remains to be established in prospective comparative studies. In endemic regions, mNGS should be considered early in the evaluation of children with unexplained HLH. For patients with neurological signs or severe disease, CSF analysis with mNGS and cytokine profiling may be useful to reveal central nervous system involvement and guide therapy, but this recommendation is based on a limited number of cases and requires further validation.

## Data Availability

The raw data supporting the conclusions of this article will be made available by the authors, without undue reservation.
