# Supplementary material for: Scrub typhus triggers hemophagocytic lymphohistiocytosis in children: metagenomic next-generation sequencing enables rapid diagnosis
Source: Front Public Health. 2026 Sep 16;14:1888645. doi: 10.3389/fpubh.2026.1888645 (PMC13623907; doi:10.3389/fpubh.2026.1888645)
Supplement: Supplementary file 1 [file Table_1.docx]

**Table S1. Baseline characteristics of scrub typhus patients without HLH (n=10)**

| Characteristic | Value |
| --- | --- |
| Median age, years (range) | 6.5 (1.5–14.0) |
| Male/Female | 4/6 |
| Median fever duration before admission, days (IQR) | 5 (4–7) |
| Eschar present, n (%) | 5 (50.0%) |
| Lymphadenopathy, n (%) | 7 (70.0%) |
| Hepatomegaly, n (%) | 2 (20.0%) |
| Splenomegaly, n (%) | 3 (30.0%) |
| Received empirical doxycycline/azithromycin, n (%) | 10 (100%) |
| Required ICU admission, n (%) | 1 (10.0%) |
| Median hospital stay, days (IQR) | 6 (4–9) |
| Survived, n (%) | 10 (100%) |

All 10 patients were diagnosed clinically based on fever, eschar, and/or epidemiological exposure, and responded to anti-rickettsial antibiotics. None met HLH-2004 diagnostic criteria. mNGS was not performed in these patients, as they were diagnosed empirically and improved with standard treatment.
